# Effect of implant placement depth on the peri-implant bone defect configurations in ligature-induced peri-implantitis: An experimental study in dogs

**DOI:** 10.4317/medoral.22032

**Published:** 2017-12-24

**Authors:** Baoxin Huang, Li Zhang, Li Xu, Weidong Zhu, Lukasz Witek, Nick Tovar, Paulo G. Coelho, Huanxin Meng

**Affiliations:** 1 PhD, Department of Oral Implantology, Guanghua School of Stomatology, Hospital of Stomatology, Sun Yat-sen University, Guangzhou, China; 2 PhD, Guangdong Provincial Key Laboratory of Stomatology, Guangzhou, China; 3 PhD, Department of Periodontology, Peking University School and Hospital of Stomatology, Beijing, China; 4 MS, PhD, Department of Biomaterials and Biomimetics, New York University College of Dentistry, NY, USA; 5 DDS, PhD, Hansjörg Wyss Department of Plastic Surgery, New York University School of Medicine, New York, NY USA

## Abstract

**Background:**

The subcrestal placement of implant platform has been considered a key factor in the preservation of crestal bone, but the influence of implant placement depth on bone remodeling combined with peri-implantitis is not fully understood. The aim of this study was to assess the effect of the crestal or subcrestal placement of implants on peri-implant bone defects of ligature-induced peri-implantitis in dogs.

**Material and Methods:**

Eight weeks after tooth extraction in six beagle dogs, two different types of implants (A: OsseoSpeed™, Astra, Mölndal, Sweden; B: Integra-CP™, Bicon, Boston, USA) were placed at either crestal or subcrestal (-1.5 mm) positions on one side of the mandible. Ligature-induced peri-implantitis was initiated four weeks after the installation of the healing abutment connections. After 12 weeks, tissue biopsies were processed for histological analyses.

**Results:**

Supra-alveolar bone loss combined with a shallow infrabony defect was observed in crestal level implants while deep and wide infrabony defects were present in subcrestal level groups. Subcrestal groups showed significantly greater ridge loss, depths and widths of infrabony defects when compared to crestal groups (*P*<0.001).

**Conclusions:**

Within the limitations of the animal study, it can be stated that the implants at subcrestal position displayed greater infra-osseous defect than implants at crestal position under an experimental ligature-induced peri-implantitis.

** Key words:**Subcrestal, peri-implantitis, histology.

## Introduction

Subcrestal implant placement in esthetic areas has been a common treatment modality in order to maintain the mucosa texture and tonality, as well as provide sufficient space to achieve an ideal emergence profile (1,2). Meanwhile, data from biomechanical analysis have indicated that increased implant placement depth could reduce the strain levels in peri-implant bone (3). Different types of implant-abutment connections have indicated different patterns of bone loss. Compared to external connections and internal screwed flat connection, conical internal connection has exhibited higher stability (4), improving resistance to micro-movement, reducing bacterial microleakage and preventing the loss of crestal bone.

Animal models using implants with morse tapered implant-abutment interface (IAI) have previously indicated a positive impact on bone contact with the neck of the implant when positioned at a subcrestal level (2,5-7). However, clinical studies utilizing implants with tapered internal IAI inserted at subcrestal levels presented contradictory results with respect to peri-implant bone loss (8-12). In a retrospective study, Lee *et al.* showed that the failure rate for the implants placed at the margin level was significantly greater than implants placed ~2mm subcrestally (8). Conversely, results from a 36-month prospective split-mouth clinical trial (9) and a 3-month prospective randomized controlled clinical trial (10) indicated no statistically significant differences in crestal bone loss around implants placed at crestal and subcrestal levels. Moreover, results from a prospective 60-month follow-up study showed peri-implant bone loss was significantly greater in subcrestal implants with platform-switched morse taper connection (11,12).

Previous studies have documented greater peri-implant probing depth, biologic width and epithelial dimension around subcrestal implants compared to crestal implants or super-crestal implants (5,6,13). It has been shown that the dental hygiene prophylaxis played an important role in maintaining the soft tissue and crestal bone levels around subcrestal implants (9). Long-term bone levels around dental implants are maintained with proper oral hygiene (14). Since peri-implant inflammation induced by poor plaque control might compromise the success of dental implants, it was of interest whether implant placement depth would affect the peri-implant bone remodeling during the development of peri-implant infections. Despite the favorable effect of the morse-tapered IAI connection, limited information is available about whether different morse-tapered IAI connections result in different peri-implant bone loss. To the best of our knowledge, no study currently exists comparing the histological bone loss between tapped-in morse-taper IAI and screwed-in morse-taper IAI at crestal and subcrestal positions under inflamed condition.

The experimental peri-implantitis model in dogs was widely used for evaluating the pathogenesis of peri-implantitis, in which the bone defect configurations was similar to the naturally occurring bone defects in humans (15). Therefore, the primary aim of this study was to histologically evaluate the effect of insertion depth on peri-implant bone defects under ligature-induced peri-implantitis in a canine model. While the secondary aim was to explore potential differences in bone defect configurations due to the implant type, tapped-in morse-taper IAI or screwed-in morse-taper IAI. The null hypothesis stated that the vertical positioning of implant along with the IAI connection would not affect peri-implant bone defect configurations in ligature-induced peri-implantitis.

## Material and Methods

-Animals

Ethics approval was obtained by the Medical Ethical Committee for Animal Investigations of Peking University Health Science Center in Beijing, China, registered under number LA2010–032 and all procedures were done according to the ARRIVE guidelines (16). Upon receiving approval, six male beagle dogs, 1-2 years old and weighting 10-12.5kg, were acquired and housed individually in standard cages under ambient temperature 20–25 ◦C, relative humidity 30–70%. All dogs were fed a soft diet and water ad libitum during the experiment. All surgical procedures were performed under general anesthesia, using intravenous sodium pentobarbital (30mg/kg). Sample size was based on the calculation of a mean difference of 1.0 mm in infra-osseous defect between groups, SD 0.6 mm, significance level of (α) 5% and 80% power.

-Study design

**Figure 1:**
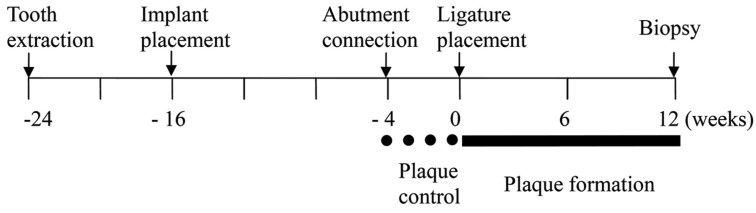
Outline of the study.

The outline of the experiment is presented in Figure 1 with the study consisting of three experimental phases. In phase 1, mandibular premolar and molar (P2-M1) were extracted bilaterally. After eight weeks of healing, a twenty-four (N=24) titanium implants were placed with a predetermined random sequence at the four experimental sites in one side of the mandible (n = 4 implants per animal) and the implants were submerged for 12 weeks (Phase 2). Four weeks after the abutment connection, oral hygiene procedures were purposefully neglected and ligature-induced experimental peri-implantitis was initiated (Phase 3). All animals were euthanized according to the protocol after 12 weeks.

Two of each implant, screwed-in tapered internal IAI and fluoride-modified TiOblast surface (A) (OsseoSpeed, 3.5 × 8 mm; Astra Tech Dental, Mölndal, Sweden) and tapped-in tapered internal IAI and plasma-sprayed calcium-phosphate surface (B) (Integra-CP, 3.5 × 8 mm; Bicon Dental Implants, Boston, Massachusetts, USA) were inserted in one side of the mandible of each animal (N=24 implants). The study consisted of four experimental groups: (1) A placed crestally (AC); (2) B placed crestally (BC); (3) A placed 1.5mm subcrestally (AS); (4) B placed 1.5mm subcrestally (BS).

-Experimental procedures 

During the first surgical procedure, after general anesthesia, a local anesthesia by 2% lidocaine hydrochloride with epinephrine at 1:100,000 was administered prior to any extraction. Roots of P2-M1 were extracted individually after they were sectioned in the buccolingual direction. Resorbable 4-0 sutures (VICRYL, Ethicon, Johnson & Johnson, Langhome, PA) were used to suture the flaps and an antibiotic (penicillin G procaine 40,000 IU/kg, intramuscular) and analgesic were administered once every 24 hours for 7 days after extraction. The wound areas were cleaned daily during the first week after surgery with a 0.12% chlorhexidine solution. After eight weeks, implant surgery was performed; full-thickness mucoperiosteal flaps were raised in the mandible, the ridge was flattened under copious irrigation with sterile saline, and osteotomies were prepared according to manufacturers’ recommendation. Meticulous care was taken to maintain a ~10mm distance between dental implant centers. Each implant type, A and B were placed at crestal and subcrestal (~1.5 mm) position on one side of the mandible of each dog. Anterior and posterior positions between implant systems were interpolated to avoid any site bias while the anterior and posterior positions of crestal and subcrestal groups within same implant system were assigned at random. Cover screws and/or plug inserts of respective implant manufacturer were placed. The flaps were sutured with 4-0 nylon sutures and the sutures were removed after 10 days. Antibiotic and analgesic was administered as aforementioned.

After 12 weeks of healing the implants were surgically uncovered. The cover screws were removed and replaced by healing abutments. Special attention was taken to avoid any occlusal contact. Ten days after the procedures, implant sites were irrigated with 0.12% chlorhexidine every second day. Subsequently, a plaque control program, which included the cleaning of implants and teeth using a toothbrush every second day was initiated.

-Experimental peri-implantitis

Four weeks after the abutment placement, experimental peri-implantitis was initiated. Oral hygiene procedures were neglected and cotton ligatures were placed sub-marginally around the abutments to facilitate plaque accumulation and to induce plaque-associated peri-implant inflammation. Ligatures were examined once a week without forcing them into an apical position. Plaque accumulation continued for a 12-week period.

-Histological preparation

Twelve weeks after ligature placement, dogs were euthansized and samples retrieved en bloc for histologic and histomorphometric analyses. Sacrifice was performed under general anesthesia by over-dose via intravenous injections of sodium pentobarbital and perfused through the carotid arteries with 4% formaldehyde. The mandibles with the implants were remove and initially fixed in 4% formaldehyde solution, which then were block-resected using an oscillating saw such that the peri-implant mesial and distal soft tissues remained intact. Gradual dehydration was accomplished using a series of alcohol solutions (70-100%). Subsequently, samples were embedded in a methacrylate-based resin (Technovit 9100, Heraeus Kulzer GmbH, Wehrheim, Germany) for non-decalcified sectioning. From each implant site, one buccal–lingual section and one distal section (~300μm thickness) was obtained and further reduced to a final thickness of about ~30μm by means of a series of SiC abrasive papers in a polishing machine under water irrigation. The buccal–lingual sections were stained in toluidine blue and the distal sections were stained with a Goldner trichromic staining for the visualization of soft tissue.

-Histomorphometric analysis

**Figure 2:**
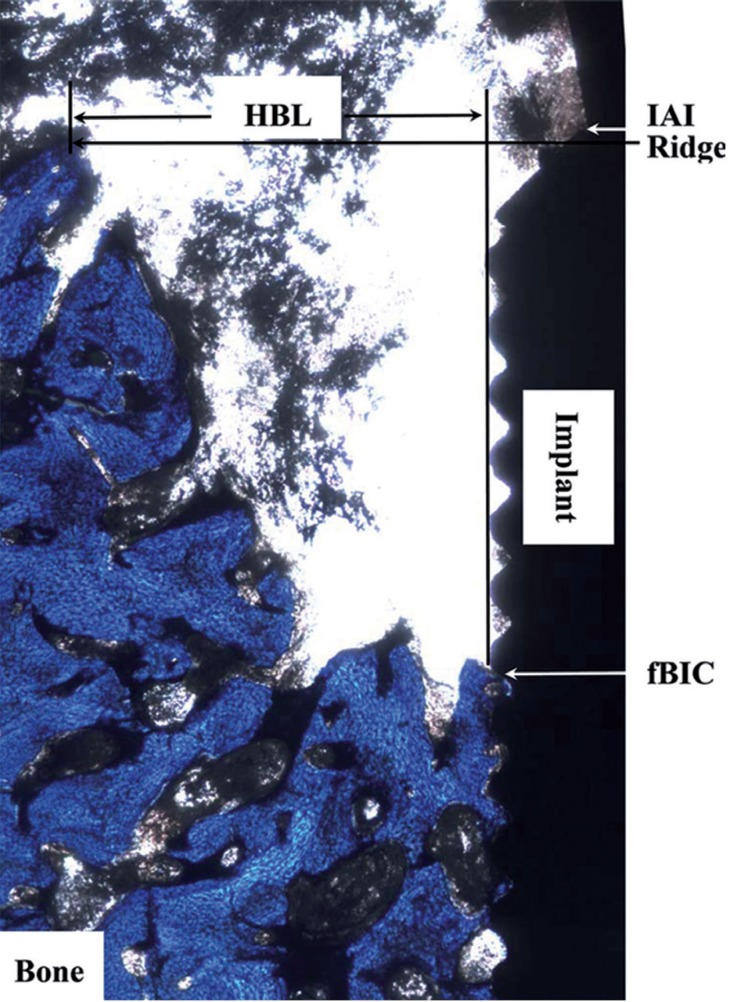
The landmarks for the measured histologic parameters: (A) Ridge; (B) IAI: implant-abutment interface; (C) fBIC: first bone-implant contact; and (D) HBL: horizontal bone loss.

All sections were referred to optical microscopy for histomorphologic evaluation. Slides had the following landmarks identified (Fig. 2): IAI, implant-abutment interface; fBIC, first bone-to-implant contact, and Ridge, the bone crest. The parameters assessed were: (1) vertical bone loss, linear distance from IAI to fBIC (IAI-fBIC); (2) ridge loss: the ridge loss was calculated as Ridge-IAI + initial insertion depth (i.e. 0 or +1.5mm); (3) depth of infrabony defect, linear distance from ridge to the fBIC (Ridge-fBIC); (4) horizontal bone loss (HBL), linear distance from Ridge to the implant body. Buccal, lingual and distal bone remodeling was measured independently.

Configuration assessment of bone defects was performed basing on the buccal–lingual sections, distal sections and the X-ray evaluation without open flap surgery. The classification of peri-implant bone defects was according to the descriptions of Schwarz *et al.* (15).

Morphometrical analyses were performed by one calibrated examiner (BH), who was not blinded due to the nature of the study. Before the analyses, a calibration procedure was initiated and revealed that repeated measurements of n = 6 different sections were similar at >95% level.

-Statistical analysis

The SPSS software (SPSS 18.0, Chicago, IL, USA) and the R software (version 3.0.1; R foundation for Statistical Computing, Vienna, Austria) were used for statistical analysis. Using the implant as the statistical unit (n = 6), the mean values, standard deviations, and median for each variable was calculated for each implant in each animal. The R-library “nparLD 2.1” (17) was used to perform the Brunner–Langer nonparametric analysis of longitudinal data in factorial experiments. Effects of IAI placement depth, implant type, and their interaction on all parameters were assessed. The alpha (α) error was set at 5%.

## Results

-Clinical findings

Healing was uneventful for all implants. Clinically, plaque accumulation was associated with hyperplasia and redness of the mucosa after ligature-induced plaque formation. The marginal alveolar bone loss was confirmed by radiographic evaluation.

-Histological evaluation

**Figure 3:**
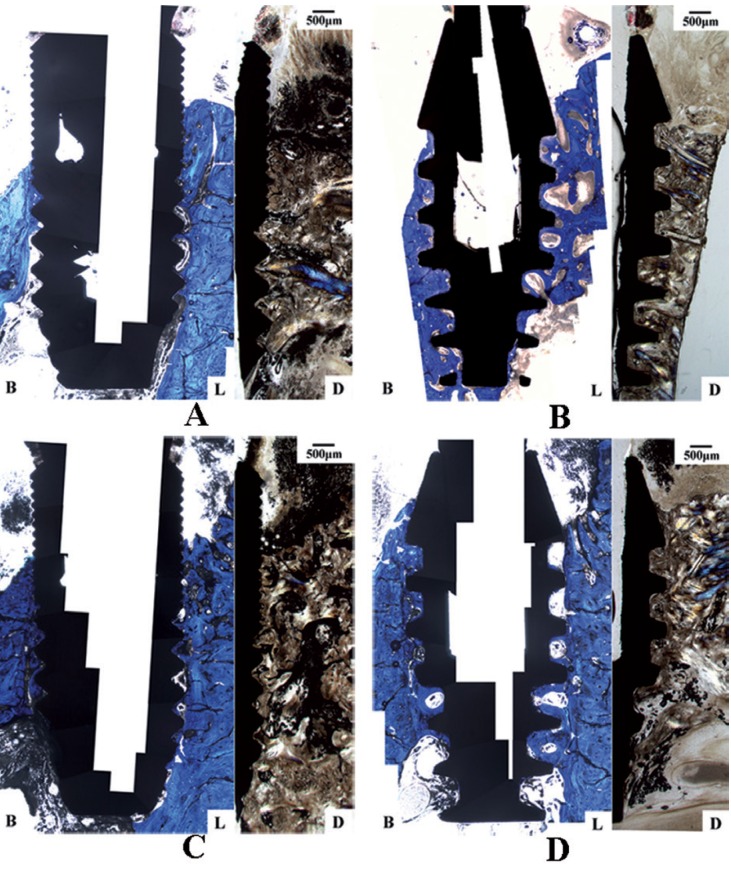
Histological sections illustrating the bone defects at (A) Astra implant placed crestally; (B) Bicon implant placed crestally; (C) Astra implant placed 1.5mm subcrestally; and (D) Bicon implant placed 1.5mm subcrestally. B and L, Buccal and lingual (toluidine blue stain); D, distal (Goldner trichromic stain).

Supra-alveolar bone losses were seen in all planes of the sections. In buccal aspects, supra-alveolar bone losses were prominent and majority of implants (20/24 implants) presented supra-alveolar bone loss without infrabony defect (Fig. 3). In the lingual and distal aspects, supra-alveolar bone losses were less pronounced compared to the buccal aspects (*P* < 0.05). The buccal orientation had significantly larger IAI-fBIC in comparison to lingual and distal orientation (*P* < 0.001), and the lingual and distal orientation did not result in a significant difference (*P* > 0.05). As a result, lingual and distal measurements of each implant were averaged for using in the analyses comparing implant subgroups. Results of the histometric measurements are presented in  Table 1. In the results of ANOVA-Type Statistic for IAI-fBIC, ridge loss, Ridge-fBIC and HBL ( Table 2), there was no significant interaction between IAI placement depth and implant type (*P* > 0.05).

**Table 1:**
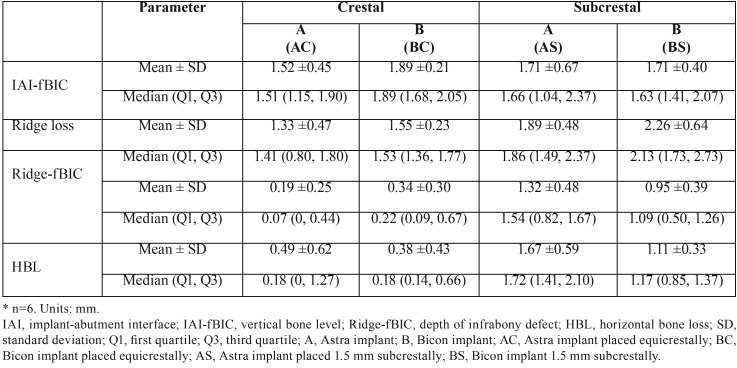
Descriptive statistics for measured outcomes*.

**Table 2:**
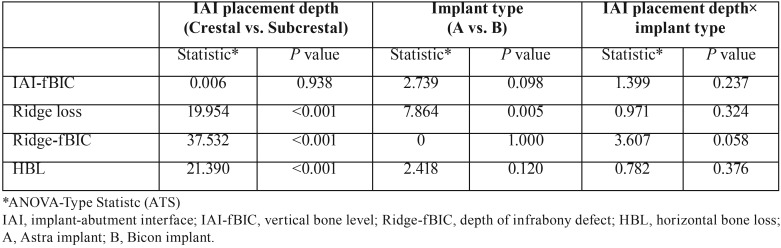
Effects of IAI placement depth, implant type, and their interaction on peri-implant bone remodeling.

Regarding bone defect configurations, frequency distributions of Class Ia–e and Class II defects (ridge to IAI) in four groups are summarized in  Table 3. In particular, 50% of implants in subcrestal groups presented without Class II defects due to the nature of subcrestal placement of IAI, even though the ridge loss was more pronounced. Bone defects were most frequently of Class Ic (75%) and following by Class Ie (25%) in subcrestal groups. In crestal groups, bone defects were most frequently of Class Ic (50%) and following by Class Ib (33%). Classes Id was not observed.

**Table 3:**
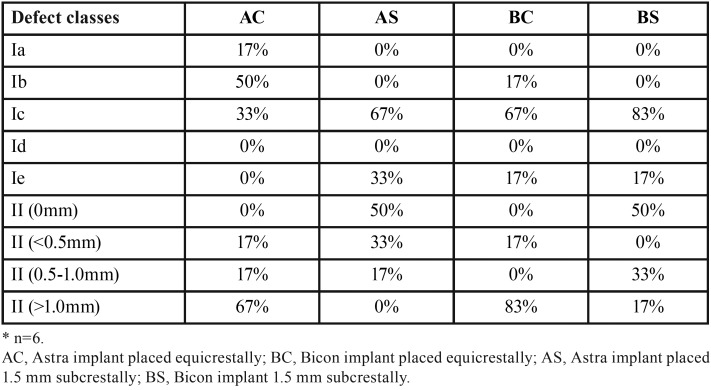
Frequency distribution of different defect classes*.

The main effect of IAI placement depth was significant for ridge loss, Ridge-fBIC and HBL values (*P* < 0.001, *P* < 0.001 and *P* < 0.001, respectively), with mean ridge loss, depths of infrabony defect (Ridge-fBIC), and the widths of infrabony defect (HBL) significantly greater for the subcrestal groups compared to the crestal groups. The main effect of IAI placement depth was not significant for vertical bone loss (IAI-fBIC) (*P* =0.938).

The main effect of implant type was significant for ridge loss (*P* = 0.005), with mean ridge loss significantly greater for B implants compared to A implants. The main effect of implant type was not significant for vertical bone loss (IAI-fBIC), depths of infrabony defect (Ridge-fBIC), and the widths of infrabony defect (HBL) (*P*=0.098, *P* =1.000 and *P* = 0.120, respectively).

## Discussion

In the present study, peri-implant bone defect around crestal implants and subcrestal implants subjected to ligature-induced peri-implantitis was analyzed. The results indicated that, irrespective of the implant type, implant placement depth had a significant effect on peri-implant bone defect configurations under experimentally induced peri-implantitis. When compared with the crestal groups, depth and width of peri-implant infrabony defect were significant greater in subcrestal implants.

In this study, peri-implantitis was induced by ligature in dogs, which is a useful model for evaluating the pathogenesis of peri-implantitis (18). Subgingival bacterial accumulation after ligature placement led to soft tissue inflammation and bone loss. Available evidence indicated that the ligature-induced peri-implantitis bone defects in dogs were comparable with naturally occurring lesions observed in humans (18).

With respect to the peri-implant bone defect configurations, the ridge loss, depth of infrabony defect (Ridge-fBIC) and the width of infrabony defect (HBL) around the subcrestal implants were significantly greater than the crestal level implants. Despite a more pronounced ridge loss, the subcrestal positioning of the implant helped maintain the ridge at the IAI level. Therefore, supra-alveolar bone loss combined with shallow circumferential infrabony defect was frequently observed in crestal implants while deep and wide infrabony defects were present in subcrestal implants. Compared to other studies, which inserted implants at crestal level under ligature-induced peri-implantitis (19,20), the depths and widths of infrabony defect obtained in this study show similar results. More marked depths and widths of infrabony defect around the subcrestal implants may due to two principal reasons. First, subcrestal implants were placed in more apical position initially, which lead to more advanced bone defect before ligature placement (5). This speculation is in agreement with the results of a systematic review, which concluded that subcrestal positioning of the IAI was associated with a higher net bone loss compared to implants placed in crestal position (21). However, it is not the only reason to explain the result of present study, as one should note that the subcrestal positioning of morse-taper IAI may help retain the bony coverage of the rough surface under non-inflamed conditions, which led to significant lower IAI-fBIC (2,5-7). As previous studies have shown, subcrestal positioning of morse-taper IAI accompany with narrow HBL (22). In the present study, the IAI-fBIC around subcrestal implants was comparable to the -crestal implant group with more significant HBL being present in subcrestal implant group. Furthermore, bone loss was more pronounced in subcrestal implants compared to crestal implants during the period of ligature-induced peri-implant infection (23). It may be attributed to the epithelium in subcrestal implants were larger than that in crestal implants (5,6), which led to more significant peri-implant probing depth after ligature-induced plaque accumulates (23). Previous studies indicated that there was a positive correlation between the peri-implant probing depth and the level of periodontal pathogens (24), and the quality and quantity of the bacterial attacks were related to the severity of peri-implant destruction (25).

The results of the present study are in agreement with the results of a recent prospective clinical study by Cassetta *et al.* (11) who inserted 576 implants in 270 patients and took the peri-apical radiographs at prosthetic loading and 60 month follow-up. They reported that peri-implant bone loss was significantly higher in subcrestal implants with platform-switched morse taper connection. Unfortunately, the information of peri-implant soft tissue parameters such as plaque index, gingival index and probing depth were lacking, which makes it difficult to judge whether the oral hygiene had any effect. In contrast, the 36-month results from a prospective split-mouth clinical trial (9) showed that crestal bone loss around platform-switched implants placed at subcrestal levels were similar with implants placed at crestal levels under well oral hygiene maintenance. This data together with the results of the present study, indicate that implants inserted at the subcrestal position can function well in a healthy condition; however, in case of subgingival plaque accumulation, the bone defect seems to be different when compared with implants inserted in the crestal position. From a clinical perspective, the pattern of the bone defect may affect the approach and potential outcome of peri-implantitis treatment (26).

Upon further evaluation of the results, it was observed that both commercial implant types had similar bone defect configurations under inflammation, except the ridge loss was greater in the B implant group. The difference in ridge loss should be interpreted with caution because previous study indicated greater affinity to ridge loss with B implants compared to the A implants prior to placement of ligature (23). No differences were found between the two different implants in terms of depths and widths of infrabony defect, although differences between the two implants included implant-abutment connection, neck shape, implant surface characteristics and thread design.

It should also be noted that the present animal trial had several limitations. Firstly, in contrast to the animal model using spontaneous progression of ligature-induced peri-implantitis, this study utilized a ligature-induced prei-implantitis model. Therefore, the influence of ligatures cannot be entirely excluded. To minimize the influence of the ligatures, they were maintained without changing or adding during the experimental period, which reduced traumatic influence on the surrounding tissues and decreased the influence of the operator on the location of the ligature. Secondly, all implants were evaluated under unloaded conditions. Previous Study reported that bone resorption was more severe when the implant was overloaded in the presence of plaque-induced inflammation (27). Despite its limitation and its preliminary character, this study indicates that shape of peri-implantitis bone defects was influenced by the depth of implant placement. Subcrestal implants showed a significant infrabony defect while crestal implant presented supra-alveolar bone loss combined with shallow infrabony defect.

## Conclusions

Within the limits of this study, it is concluded that implants placed at the subcrestal position displayed greater infra-osseous defects than those implants placed at the crestal position in a ligature induced peri-implantitis model.
